# Elevated Urotensin-II and TGF-β Levels in COPD: Biomarkers of Fibrosis and Airway Remodeling in Smokers

**DOI:** 10.3390/medicina60111750

**Published:** 2024-10-24

**Authors:** Metin Kilinc, Ibrahim Demir, Semih Aydemir, Rauf Gul, Recep Dokuyucu

**Affiliations:** 1 Department of Anesthesiology and Reanimation, Faculty of Medicine, Mardin Artuklu University, Mardin 47200, Turkey; metinkilinc@artuklu.edu.tr; 2 Department of Anesthesiology and Reanimation, Mardin Training and Research Hospital, Mardin 47000, Turkey; dribo21@hotmail.com; 3 Department of Anesthesiology and Reanimation, Yıldırım Beyazit University Yenimahalle Training and Research Hospital, Ankara 06370, Turkey; drsemihaydemir@gmail.com; 4 Department of Anesthesiology and Reanimation, School of Medicine, Gaziantep University, Gaziantep 27410, Turkey; gulrauf@gantep.edu.tr; 5 Department of Physiology, Medical Specialization Training Center (TUSMER), Ankara 06420, Turkey

**Keywords:** COPD, fibrosis, pathophysiology, urotensin II, transforming growth factor-β

## Abstract

*Background and Objectives:* Small airway fibrosis plays a critical role in the progression of chronic obstructive pulmonary disease (COPD). Previous research has suggested that Urotensin-II (U-II) and transforming growth factor-β (TGF-β) may contribute to pathological fibrosis in various organs, including the cardiovascular system, lungs, and liver. However, their specific relationship with airway fibrosis in COPD has not yet been thoroughly investigated. This study aims to evaluate the concentrations of U-II and TGF-β in individuals with COPD, as well as in healthy smokers and non-smokers, to explore their potential roles in COPD-related fibrosis. *Materials and Methods:* The study included three distinct groups: a healthy non-smoker control group (n = 98), a healthy smoker group (n = 78), and a COPD group (n = 80). All participants in the COPD group had a smoking history of at least 10 pack-years. COPD was defined according to the Global Initiative for Chronic Obstructive Lung Disease (GOLD) guidelines, with only patients classified as GOLD stage 2 or higher being included in the study. Urotensin-II (U-II) and transforming growth factor-β (TGF-β) levels were measured using a commercially available ELISA kit. *Results:* COPD patients had a significantly lower FEV_1_ (58 ± 15.4%) compared to smokers (79 ± 4.5%) and non-smokers (92 ± 3.7%) (*p* < 0.001). Similarly, COPD patients had a lower FEV_1_/FVC ratio (55 ± 9.4%) compared to smokers (72 ± 4.2%) and non-smokers (85 ± 3.6%) (*p* < 0.01 and *p* < 0.05, respectively). SaO_2_ was significantly lower in COPD patients (87%) compared to smokers (96.5%) and non-smokers (98%) (COPD vs. smokers: *p* < 0.05 and smokers vs. non-smokers: *p* > 0.05). U-II levels were significantly higher in COPD patients (175.10 ± 62.40 pg/mL) compared to smokers (118.50 ± 45.51 pg/mL) and non-smokers (85.29 ± 35.87 pg/mL) (*p* < 0.001 and *p* < 0.05, respectively). COPD patients also had significantly higher levels of TGF-β (284.60 ± 60.50 pg/mL) compared to smokers (160.00 ± 41.80 pg/mL) and non-smokers (92.00 ± 25.00 pg/mL) (*p* < 0.001 and *p* < 0.05, respectively). *Conclusions:* Our study supports the growing body of evidence that U-II and TGF-β play central roles in the development and progression of fibrosis in COPD. The negative correlation between these markers and lung function parameters such as FEV_1_ and FEV_1_/FVC indicates that they may be key drivers of airway remodeling and obstruction. These biomarkers could serve as early indicators of fibrotic changes in smokers, even before the onset of COPD.

## 1. Introduction

Chronic obstructive pulmonary disease (COPD) is one of the leading causes of morbidity and mortality worldwide, contributing significantly to the global burden of disease [1]. It is characterized by persistent airflow limitation and chronic inflammation, leading to progressive respiratory symptoms such as dyspnea, chronic cough, and sputum production. The pathogenesis of COPD involves a complex interplay of genetic, environmental, and inflammatory factors that result in airway remodeling, tissue destruction, and increased susceptibility to infections. Beyond the physical impact on patients, COPD imposes a substantial economic burden due to the high costs associated with its management, including frequent hospitalization, long-term pharmacotherapy, and the need for respiratory support [2]. In addition to its economic toll, COPD significantly affects community healthcare systems by contributing to physical disability and impaired quality of life among patients. These limitations often result in a decreased ability to perform daily activities, increased dependency, and reduced social participation, further exacerbating the overall societal and healthcare costs [3].

Although smoking is the most important and well-established risk factor for the development of COPD, this condition can also occur in individuals who have never smoked, highlighting the multifactorial nature of its etiology [4,5,6]. Other risk factors contributing to COPD include genetic predispositions, such as the imbalance between proteolytic and anti-proteolytic enzymes; oxidative stress resulting from environmental exposures (e.g., air pollution, occupational hazards); and systemic inflammation, all of which play a significant role in the disease’s development and progression [6,7,8,9,10]. These factors can cause widespread damage to lung structures, affecting not only the pulmonary parenchyma, but also the pulmonary vascular system, as well as the proximal and peripheral airways [11,12]. Pathologically, COPD is characterized by chronic inflammation in these regions, with an increased number of specific inflammatory cells, including neutrophils, macrophages, and lymphocytes. Over time, this inflammation leads to structural remodeling, a process that involves tissue damage and attempts at repair in various areas of the lungs [13]. This continuous cycle of injury and repair results in permanent structural changes, such as airway narrowing, destruction of alveolar walls (emphysema), and increased airway resistance, all of which contribute to the progressive nature of the disease. One of the hallmark features of COPD is small airway fibrosis, which plays a crucial role in airflow limitation. The fibrosis is believed to be the result of chronic inflammation, which drives fibroblast activation. Fibroblasts, which are responsible for producing extracellular matrix proteins like collagen, become activated in response to various fibrogenic factors such as Transforming Growth Factor-β (TGF-β), connective tissue growth factor (CTGF), and endothelin, which are secreted by epithelial cells and macrophages in the lungs [14,15]. Importantly, the extent of inflammation and structural remodeling in the respiratory tract is closely associated with disease severity. Even after smoking cessation, which is the most effective intervention to slow disease progression, these pathological changes can persist, and in some cases, continue to worsen. This underscores the need for effective anti-inflammatory treatments aimed at halting or reversing fibrosis and preventing further lung damage [16]. Addressing the inflammatory and fibrotic components of COPD could improve disease outcomes, reduce the progression of airway remodeling, and enhance the quality of life of patients.

Urotensin II (U-II) is a novel peptide that is widely distributed throughout various tissues and has both physiological and pathological effects on multiple organ systems. It is considered one of the most potent endogenous vasoconstrictors known to date, exerting significant influence over vascular tone and blood pressure regulation. U-II exerts its effects through binding to its specific receptor, the human orphan G-protein-coupled receptor (GPR14), which is highly expressed in vascular smooth muscle cells, endothelial cells, and various other tissues. Despite its well-recognized role in vasoconstriction, the precise metabolic pathways and broader physiological implications of U-II remain incompletely understood [17,18,19]. In addition to its vasoactive properties, U-II has been implicated in the development of fibrotic changes, particularly in cardiovascular and pulmonary tissues. Angiotensin II and Endothelin-1 are well-established mediators of cardiac fibrosis, contributing to the progression of heart failure through the promotion of extracellular matrix deposition and tissue remodeling [20]. Research has demonstrated that U-II can activate TGF-β in cardiac fibroblasts, leading to increased collagen production and extracellular matrix accumulation, both of which contribute to the fibrotic remodeling of the myocardium [21]. For instance, a study by Onat et al. highlighted the role of U-II in lung fibrosis, showing that U-II induces fibrotic changes in lung tissue through the upregulation of TGF-β, thus promoting fibroblast activation and collagen deposition [22]. Similarly, Zhang et al. found that elevated levels of U-II are associated with increased TGF-β production, further supporting the link between U-II and fibrotic pathways [23]. Moreover, other studies have corroborated these findings, reporting that U-II significantly increases the levels of TGF-β in various tissues, although the precise molecular mechanisms by which U-II stimulates TGF-β production remain unclear [21,24,25,26].

We think that U-II and TGF-β contribute to the progression of COPD and fibrosis by triggering oxidative stress. This may be related to inflammation mediated by NADPH, which is released by the airway epithelium. In addition, remodeling occurs during the pathogenesis of COPD, and one of the most important reasons for this is fibrosis. This study aimed to compare U-II and TGF-β levels among COPD patients, smoking healthy individuals, and non-smoking healthy subjects.

## 2. Materials and Methods

### 2.1. Study Design and Study Population

The study protocol received approval from the local ethics board at Mustafa Kemal University. Additionally, informed consent was obtained from all 256 participants enrolled in the study. Of these, 98 were healthy non-smokers (female: 49, male: 50, mean age: 43.88 ± 13.37), 78 were healthy smokers (female: 29, male: 49, mean age: 40.33 ± 13.12), and 80 had COPD (female: 48, male: 32, mean age: 59.04 ± 11.87). Healthy smokers and non-smokers were recruited from the general population, including hospital staff and community volunteers present at local health centers. COPD patients were recruited from the Pulmonology Department at Mustafa Kemal University Hospital during routine consultations. The inclusion criteria for COPD patients in this study required participants to have a confirmed diagnosis of COPD based on GOLD criteria (level 2 or higher) and a minimum smoking history of 10 pack-years [27]. The smoker group consisted of individuals with at least 10 pack-years of smoking history but no diagnosis of respiratory disease, while the non-smoker group included those with no smoking or respiratory disease history. Participants were aged 40–75 years, and informed consent was mandatory. Exclusion criteria included other respiratory diseases (e.g., asthma, bronchiectasis), significant cardiovascular diseases (e.g., heart failure, coronary artery disease), chronic inflammatory diseases, recent use of immunosuppressive therapy, active infections within the past 4 weeks, and end-stage liver or chronic kidney disease. Pregnant women were also excluded to avoid confounding due to hormonal or inflammatory changes during pregnancy.

### 2.2. Urotensin II and TGF-β Measurements

Plasma concentrations of U-II and TGF-β were measured using commercial Enzyme-Linked Immunosorbent Assay (ELISA) kits (Uscn Life Science Inc., Wuhan, P.R. China), following the manufacturer’s protocols. Blood samples were collected from all participants after an overnight fast, and plasma was separated by centrifugation at 3000 rpm for 15 min. The plasma samples were then aliquoted and stored at −80 °C until analysis to prevent degradation of the biomarkers.

For U-II, the assay employed a sandwich ELISA technique, with a detectable range of 15.6 to 1000 pg/mL. The sensitivity, defined as the minimum detectable dose, was typically below 5.9 pg/mL. TGF-β levels were also measured using a sandwich ELISA technique, with a detectable range of 1.7 to 15.4 pg/mL, and a sensitivity typically below 1.7 pg/mL. In both assays, plasma samples and standards were added to microtiter plate wells pre-coated with antibodies specific to either U-II or TGF-β. Following the addition of a biotinylated detection antibody and a streptavidin-HRP conjugate, the substrate solution was applied, producing a color change proportional to the concentration of U-II or TGF-β present in each sample. The optical density was measured at 450 nm using a microplate reader (BioTek Instruments, Inc., Winooski, VT, USA).

All assays were run in duplicate to ensure reproducibility, and the intra-assay and inter-assay coefficients of variation (CV) for U-II and TGF-β were <10% and <15%, respectively. Calibration curves were generated using the standards provided in the kit, and the concentrations of U-II and TGF-β in the plasma samples were calculated by comparing their optical density values to the standard curves. Samples with concentrations exceeding the detectable range were appropriately diluted and reassayed.

### 2.3. Statistical Analysis

Statistical analysis was performed using the Statistical Package for the Social Sciences (SPSS), version 27.0 (IBM Corp., Armonk, NY, USA). A priori power analysis was conducted using G*Power software (version 3.1.9.4). The effect size was calculated based on previous studies evaluating U-II and TGF-β in COPD populations. With a desired power of 0.80 and an alpha level of 0.05, the required sample size for detecting significant differences between groups was determined to be 75 subjects per group. The chi-square test was used for categorical variables, while the normality of continuous variables was assessed with the Kolmogorov–Smirnov test. Differences between group means were analyzed using one-way ANOVA, followed by post hoc Bonferroni tests. The relationships between continuous variables were evaluated using Spearman’s correlation coefficient. A significance level of *p* < 0.05 was considered for all statistical analyses.

## 3. Results

A comparison of the socio-demographic and laboratory data of the COPD patients, smokers, and non-smokers is shown in Table 1. Among the COPD patients, 40% were male and 60% were female. In the smoker group, 62.8% were male and 37.2% were female. In the non-smoker group, the distribution was almost equal, with 50.5% male and 49.5% female participants. There was no statistically significant difference in gender distribution among the groups (*p* > 0.05). The mean BMI was 26 ± 2.1 in the COPD group, 24 ± 1.5 in the smoker group, and 22 ± 1.3 in the non-smoker group. No significant difference was observed in BMI among the groups (*p* > 0.05). COPD patients had a significantly lower FEV_1_ (58 ± 15.4%) compared to smokers (79 ± 4.5%) and non-smokers (92 ± 3.7%) (*p* < 0.001). Similarly, COPD patients had a lower FEV_1_/FVC ratio (55 ± 9.4%) compared to smokers (72 ± 4.2%) and non-smokers (85 ± 3.6%) (*p* < 0.01 and *p* < 0.05, respectively). SaO_2_ was significantly lower in COPD patients (87%) compared to smokers (96.5%) and non-smokers (98%), with a *p*-value of < 0.05 between COPD patients and smokers, but there was no significant difference between smokers and non-smokers (*p* > 0.05). The mean age was 57.04 ± 11.87 years in the COPD group, 50.33 ± 10.12 years in the smoker group, and 53.88 ± 13.37 years in the non-smoker group (*p* > 0.05). U-II levels were significantly higher in COPD patients (175.10 ± 62.40 pg/mL) compared to smokers (118.50 ± 45.51 pg/mL) and non-smokers (85.29 ± 35.87 pg/mL) (*p* < 0.001 and *p* < 0.05, respectively). COPD patients also had significantly higher levels of TGF-β (284.60 ± 60.50 pg/mL) compared to smokers (160.00 ± 41.80 pg/mL) and non-smokers (92.00 ± 25.00 pg/mL) (*p* < 0.001 and *p* < 0.05, respectively) (Table 1).

**Figure 1:**
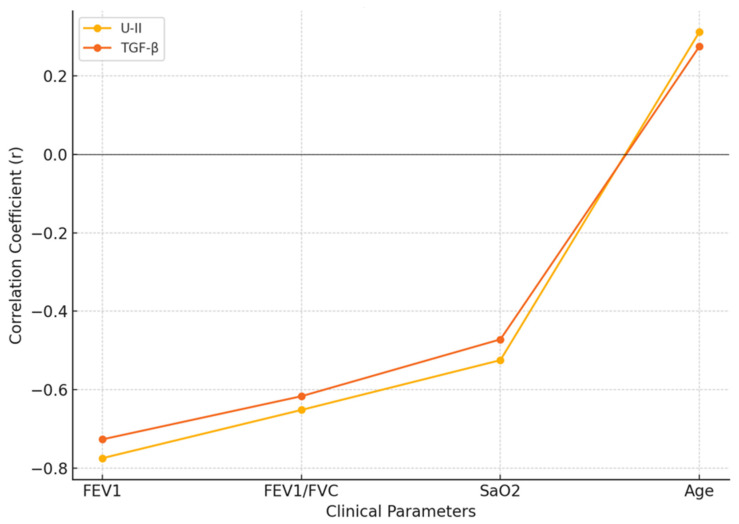
Correlation of U-II and TGF-β with clinical parameters in patients with COPD.

The correlation of U-II and TGF-β levels with various clinical parameters in the COPD group is shown in Table 2. U-II levels were negatively correlated with FEV1 (r = −0.775, *p* < 0.01), and similarly, TGF-β levels were also negatively correlated with FEV_1_ (r = −0.727, *p* < 0.01). This indicates that higher levels of both U-II and TGF-β are associated with lower lung function, as measured by FEV_1_. A negative correlation was observed between U-II and FEV1/FVC ratio (r = −0.652, *p* < 0.01), and TGF-β also showed a negative correlation with FEV_1_/FVC ratio (r = −0.617, *p* < 0.01). These results suggest that elevated U-II and TGF-β levels are associated with a decrease in the ratio of forced expiratory volume to forced vital capacity, further indicating impaired lung function. There was a significant negative correlation between U-II and SaO_2_ (r = −0.525, *p* < 0.05), as well as between TGF-β and SaO_2_ (r = −0.472, *p* < 0.05), suggesting that higher levels of these markers are linked to lower oxygen saturation in the blood. U-II levels were positively correlated with age (r = +0.312, *p* < 0.05), and TGF-β levels also showed a positive correlation with age (r = +0.275, *p* < 0.05). This indicates that older age is associated with higher levels of both U-II and TGF-β in COPD patients (Table 2, Figure 1).

## 4. Discussion

This study highlights the significant roles of U-II and TGF-β in the pathophysiology of COPD, particularly regarding their association with airway fibrosis and lung function impairment. Our findings demonstrate that COPD patients exhibit significantly higher levels of U-II and TGF-β compared to healthy smokers and non-smokers. These elevated levels are strongly correlated with decreased lung function, as indicated by lower FEV_1_ and FEV_1_/FVC ratios, as well as reduced SaO_2_.

In cardiovascular research, U-II has been shown to promote fibrosis by activating fibroblasts and enhancing collagen production, as observed by Liang et al., who demonstrated that U-II stimulates collagen synthesis via the ERK and p38 MAPK pathways in cardiac fibroblasts [21]. Similarly, in our study, COPD patients with elevated U-II levels exhibited more pronounced airflow limitation, suggesting that U-II could be promoting fibrosis in the small airways, a hallmark of COPD. The correlation we found between higher U-II levels and lower FEV_1_ suggests that U-II may contribute to airway fibrosis and, thus, the structural changes seen in advanced COPD.

TGF-β has a well-established role as a master regulator of fibrosis across multiple organ systems. Reed et al. reported TGF-β’s role in promoting the differentiation of fibroblasts into myofibroblasts, leading to excessive extracellular matrix deposition and tissue scarring [28]. In the lungs, TGF-β has been implicated in the thickening of airway walls and remodeling of lung tissue, processes that are particularly relevant in COPD [28,29,30,31]. The negative correlation between TGF-β and lung function (FEV_1_ and FEV_1_/FVC) in our study echoes the findings of Kraik et al., who reported that TGF-β signaling was upregulated in airway smooth muscle cells in COPD patients, contributing to collagen deposition and airway narrowing [32]. This suggests that the fibrotic processes driven by TGF-β are central to the progression of COPD and could be a target for therapeutic intervention.

Interestingly, Xie et al. found that TGF-β levels were higher in the bronchoalveolar lavage fluid of COPD patients with more severe disease, highlighting the localized elevation of this cytokine in the lung microenvironment [33]. Our study extends these findings by demonstrating that TGF-β is not only elevated locally, but also systemically in the serum of COPD patients. This systemic elevation may reflect a broader fibrotic response in the body, potentially affecting other organs as well, which is supported by studies showing that COPD patients often have comorbid fibrotic diseases such as pulmonary hypertension and interstitial lung disease [34,35].

Additionally, the elevated levels of U-II and TGF-β in smokers, though lower than in COPD patients, suggest that these profibrotic molecules may be activated early in the disease process, even before the onset of clinically significant airflow obstruction. Ko et al. and Chen et al. reported how chronic exposure to cigarette smoke can induce persistent inflammation in the airways, which could be activate fibrotic pathways such as those driven by U-II and TGF-β [36,37]. The subclinical fibrosis seen in smokers could, therefore, represent an early stage of airway remodeling, setting the stage for the later development of COPD. This could explain why smokers in our study had higher levels of U-II and TGF-β compared to non-smokers, although their lung function remained relatively preserved.

In contrast to other studies that focus primarily on the local effects of TGF-β in the lung, our study’s strength lies in its exploration of systemic biomarkers, providing evidence that U-II and TGF-β may not only act locally, but also serve as systemic indicators of fibrotic activity in COPD. This systemic involvement has been noted in other fibrotic conditions, such as liver fibrosis, where U-II and TGF-β are involved in stellate cell activation and collagen deposition [38,39]. Given the systemic nature of these fibrotic pathways, targeting U-II and TGF-β could potentially mitigate fibrosis not only in the lungs, but also in other affected organs.

### Limitations of the Study

Our study has several limitations that warrant consideration. First, the sample size was relatively small, particularly in subgroup analyses, which may limit the generalizability of the findings. Additionally, while we demonstrated significant associations between U-II, TGF-β, and lung function parameters, the cross-sectional design prevents us from establishing a definitive causal relationship. Longitudinal studies are needed to determine whether changes in these biomarkers precede or result from disease progression. Finally, direct assessment of airway fibrosis through imaging or histological techniques was not conducted; this would provide more robust evidence linking U-II and TGF-β to fibrotic changes in the airways.

## 5. Conclusions

In conclusion, this study demonstrates that U-II and TGF-β levels are significantly elevated in patients with COPD compared to healthy smokers and non-smokers. These findings align with previous research indicating that U-II and TGF-β are involved in fibrotic processes in various organs, including the lungs. The higher concentrations of these biomarkers in COPD patients suggest a potential role in the progression of airway fibrosis, and they may serve as potential biomarkers for assessing disease severity and progression. Further research is warranted to explore their mechanistic roles in airway remodeling and fibrosis in COPD.

## Figures and Tables

**Table 1:** A comparison of the socio-demographic and laboratory data of the participants.

	COPD (n = 80) Mean ± SD, n (%)	Smoker (n = 78) Mean ± SD, n (%)	Non-Smoker (n = 98) Mean ± SD, n (%)	*p* Value
Gender				>0.05 ^a^
Male (n)	32 (40%)	49 (62.8%)	50 (50.5%)	
Female (n)	48 (60%)	29 (37.2%)	49 (49.5%)	
BMI	26 ± 2.1	24 ± 1.5	22 ± 1.3	>0.05 ^b,c^
FEV_1_	58 ± 15.4%	79 ± 4.5%	92 ± 3.7%	<0.001 ^b^ <0.05 ^c^
FEV_1_/FVC ratio	55 ± 9.4%	72 ± 4.2%	85 ± 3.6%	<0.01 ^b^ <0.05 ^c^
Bronchodilators	20			
Bronchodilatory Therapy + Inhaled corticosteroids	60			
SaO_2_	87%	96.5%	98%	<0.05 ^b^ >0.05 ^c^
Frequency of exacerbation/year	2	-	-	
Smoking status (pack/year)	30	15	-	
Age (year)	57.04 ± 11.87	50.33 ± 10.12	53.88 ± 13.37	>0.05 ^b,c^
U-II (pg/mL)	175.10 ± 62.40	118.50 ± 45.51	85.29 ± 35.87	<0.001 ^b^ <0.05 ^c^
TGF-β (pg/mL)	284.60 ± 60.50	160.00 ± 41.80	92.00 ± 25.00	<0.001 ^b^ <0.05 ^c^

COPD: chronic obstructive pulmonary disease; BMI: body mass index; FEV_1_: forced expiratory volume in 1 s; FVC: forced vital capacity; SaO_2_: oxygen saturation; U-II: Urotensin-II; TGF-β: Transforming Growth Factor-β; ^a^ chi-square test (COPD vs. non-smoker, COPD vs. smoker); ^b^ one-way ANOVA (COPD vs. smoker, COPD vs. non-smoker); ^c^ Bonferroni test (smoker vs. non-smoker).

**Table 2:** The correlation of U-II and TGF-β levels with the parameters in the COPD group.

	U-II		TGF-β	
Variables	r Value	*p* Value	r Value	*p* Value
FEV_1_	−0.775	<0.01	−0.727	<0.01
FEV_1_/FVC ratio	−0.652	<0.01	−0.617	<0.01
SaO_2_	−0.525	<0.05	−0.472	<0.05
Ages (year)	+0.312	<0.05	+0.275	<0.05

FEV_1_: forced expiratory volume in 1 s; FVC: forced vital capacity; SaO_2_: oxygen saturation; U-II: Urotensin-II; TGF-β: Transforming Growth Factor-β.

## Data Availability

Data are available upon request to the corresponding author.
